# Enhancing teacher occupational wellbeing: the critical role of working environment in Chinese primary schools

**DOI:** 10.3389/fpsyg.2026.1817138

**Published:** 2026-05-12

**Authors:** Xue Xia, Guang Li, Hongmei Liang

**Affiliations:** Faculty of Education, Northeast Normal University, Changchun, China

**Keywords:** professional development, school culture, teachers’ occupational wellbeing, working environment, workload

## Abstract

**Background/Introduction:**

Based on the Job Demands-Resources model, this study examines how six dimensions of the working environment shape teacher occupational well-being (TOWB) in the context of high-stakes primary education systems.

**Methods:**

Drawing on a 2020 national survey of 25,028 Chinese primary school teachers, the study employed CEM and SEM to analyze the data and provide convergent evidence for causal inferences.

**Results:**

Findings reveal a moderate TOWB level with significant demographic variations. The six-dimensional working environment explains 71.6% of the variance in TOWB. Specifically, professional development and policy support are the strongest positive predictors, whereas workload exerts a robust negative impact. Furthermore, school culture partially mediates the relationship between the environment and TOWB.

**Discussion:**

The results highlight the necessity of workload audits, tailored professional development, and faithful policy implementation. This study offers contextual, methodological, and conceptual extensions of JD-R theory within the primary education sector.

## 1 Introduction

The notion that teachers’ occupational well-being (TOWB) functions as a linchpin for educational quality is widely recognized across multiple disciplinary communities. Defined as an integrated state of psychological, emotional and professional fulfilment (Wijngaards et al., 2022), TOWB not only predicts instructional excellence (Sutton and Wheatley, 2003) but also cascades into superior student academic and socio-emotional outcomes and, at the institutional level, significantly attenuates turnover intentions (Macdonald, 1999). Conversely, its erosion manifests in burnout, absenteeism and early attrition, thereby destabilizing reform trajectories (Parker et al., 2012). Such reciprocal dynamics render TOWB a strategic policy lever. Yet the mechanisms through which working environments either sustain or erode it remain under-specified, particularly in non-Western, high-stakes accountability contexts.

Since the OECD’s 2005 report “Teachers Matter: Attracting, Developing and Retaining Effective Teachers” the volume of TOWB research has grown substantially (Pei and Li, 2015). The Job Demands-Resources (JD-R) model (Demerouti et al., 2001; Hakanen et al., 2006) offers a parsimonious heuristic—posited that well-being emerges from the dynamic equilibrium between job demands that deplete energy and job resources that replenish it—its empirical applications in primary education have rarely integrated multi-dimensional environmental resources. Instead, extant studies often isolate singular factors (e.g., workload or leadership) without examining their synergistic or compensatory effects. Moreover, cultural and structural contingencies may recalibrate the salience of these demands and resources; thus, de-contextualized findings risk yielding policy prescriptions that misfire when transplanted.

The Chinese case epitomizes such contingency. Dominated by an examination-oriented culture, primary school teachers confront oversized classes (Wang and Zhang, 2017), administrative overload (Zhou and Ning, 2020a) and intensified parental expectations tied to high-stakes testing. These contextual demands are compounded by performative accountability regimes and rapid curricular reforms that reconfigure professional identities at accelerated tempos. Although prior studies have reported moderate to low levels of teacher well-being in various Chinese samples (Xin et al., 2021), no large-scale nationally representative study has systematically documented the distribution of TOWB across key demographic categories among primary school teachers This lacuna not only curtails the evidentiary basis for contextually grounded interventions but also obscures potential leverage points for sustaining teacher quality amid massification and reform imperatives.

Addressing this void, the present study adopts the updated JD-R model as an organizing scaffold and operationalizes “working environment” as a six-dimensional resource constellation: (1) physical and material conditions, (2) perceived workload manageability, (3) sustained professional development, (4) collaborative school culture, (5) participative management systems, and (6) macro policy support for work–life balance. By integrating these facets within the JD-R architecture, we seek to elucidate how specific environmental resources counteract culturally embedded job demands, thereby enhancing TOWB among Chinese primary-school teachers.

**Figure 1:**
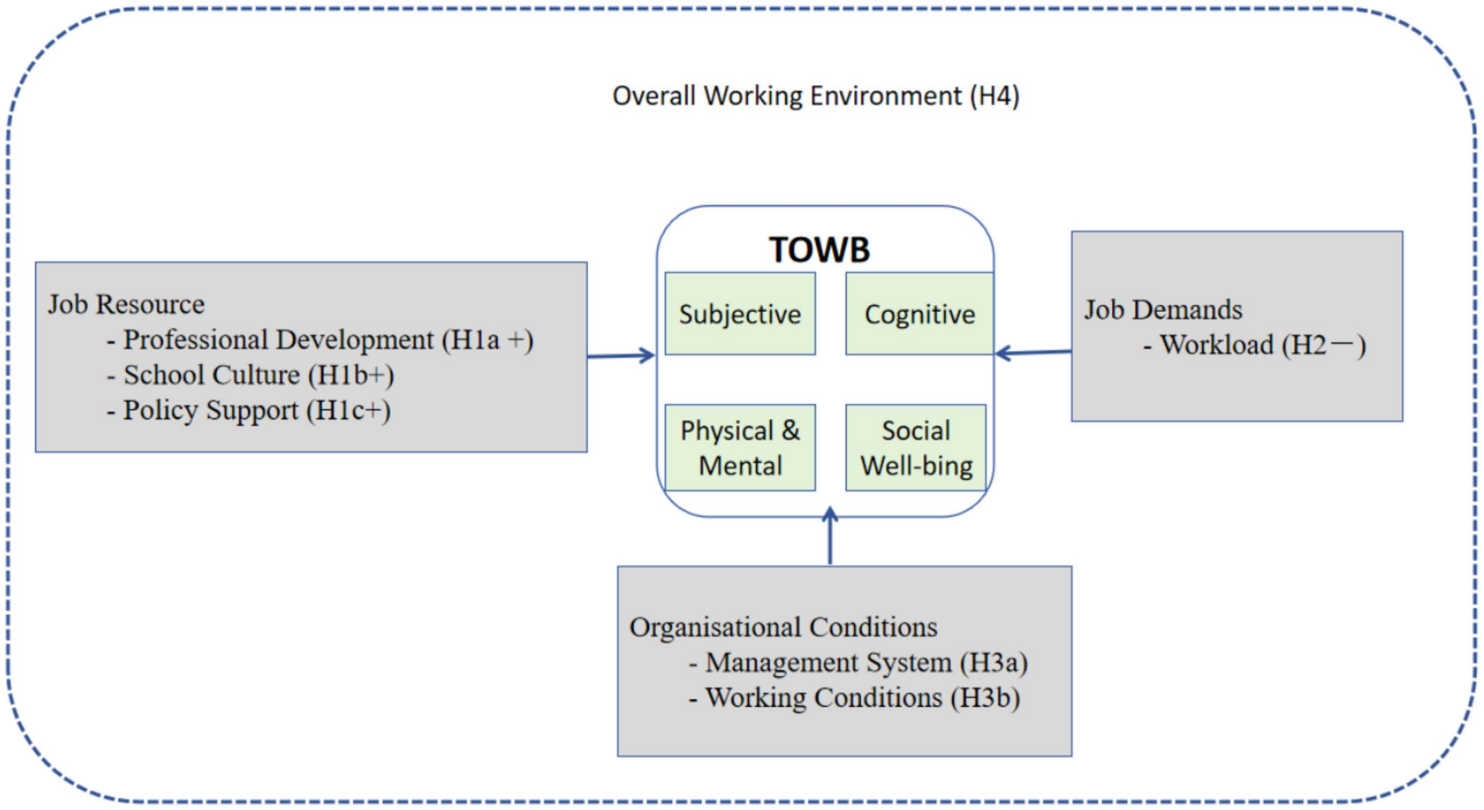
Conceptual model of the working environment and TOWB within the JD-R framework.

Figure 1 presents the conceptual model guiding this study. It depicts the six-dimensional working environment (three job resources, two job demands, and one organizational Conditions) as predictors of the four-dimensional TOWB construct (subjective, cognitive, physical/mental, and social well-being) within the JD-R framework, with demographic and school-level controls.

This study makes three distinct theoretical contributions. First, it provides a contextual extension of JD-R theory by testing a comprehensive, culturally attuned multi-dimensional operationalization of the working environment in a non-Western, examination-driven primary education system. Second, it offers a methodological advance by combining large-scale national survey data with Coarsened Exact Matching (CEM) to strengthen causal inference within cross-sectional constraints. Third, it delivers a conceptual refinement by demonstrating the synergistic buffering effects of job resources and the demand-threat mechanisms of workload in a high-accountability context, thereby clarifying boundary conditions of the JD-R model beyond Western settings.

The remainder of the paper is organized as follows. Section 2 reviews the literature and articulates the research questions. Section 3 describes the data, measures, and analytical procedures. Section 4 presents the results. Sections 5 and 6 discuss the findings, theoretical and practical implications, limitations, and directions for future research.

## 2 Literature review

### 2.1 Defining and measuring TOWB

The conceptualization of TOWB has historically been rooted in the study of job burnout, which is characterized by emotional exhaustion, depersonalization, and reduced personal accomplishment (Maslach and Jackson, 1981). This deficit-oriented approach highlighted the depletion of psychological resources under prolonged job stress. Subsequent work established burnout as a core inverse component of TOWB (Bermejo-Toro et al., 2016), positioning the two constructs as opposing poles on a well-being continuum.

The JD-R model (Demerouti et al., 2001) advanced this perspective by framing well-being as the outcome of a dynamic equilibrium. Job demands deplete energy, while job resources replenish it and buffer strain. Empirical studies have consistently shown that self-efficacy and professional identity (Brouwers et al., 2001) reduce burnout among Chinese teachers (Lu et al., 2022). Contemporary definitions have broadened TOWB beyond the absence of burnout to encompass positive psychological, emotional, and social dimensions. TOWB is now understood as the experience of positive emotions, engagement, meaning at work, and supportive relationships (Wijngaards et al., 2022). This holistic view integrates intrinsic factors with extrinsic factors (Peláez-Fernández et al., 2021), recognizing well-being as a dynamic person-context interaction rather than a static state (Hascher and Waber, 2021).

A recent meta-analysis of 58 studies confirms that JD-R antecedents explain substantial variance in TOWB across school levels, with job resources showing consistently stronger positive associations than demands show negative ones (Li et al., 2025). Yet most applications remain Western-centric or focus on secondary education, leaving primary-school teachers in high-stakes, examination-oriented systems under-examined. The present study therefore adopts a four-dimensional TOWB measure—subjective, cognitive, physical/mental, and social well-being—validated specifically for Chinese teachers (Li and Gai, 2022) to capture this multifaceted construct in context.

### 2.2 Working environment as JD-R construct

Within the JD-R framework, the working environment comprises job resources that facilitate goal attainment and personal growth while buffering demands, and job demands that require sustained physical or psychological effort (van Wingerden et al., 2017). Three resource dimensions and two demand dimensions emerge as particularly salient for TOWB.

Professional development enhances competence and mitigates burnout (Chen et al., 2022; Hollar et al., 2022). Meta-analytic evidence further shows professional development opportunities rank among the strongest predictors of teacher well-being globally (Zhou et al., 2024). In the Chinese context, however, access remains uneven, and programs often prioritize compliance over teacher agency.

School culture, encompassing collaboration, trust, and participative decision-making, functions as a social resource. Positive cultures reduce stress and enhance self-efficacy (Barbieri et al., 2019; Jones et al., 2019; Renshaw et al., 2015; Thien and Lee, 2023). Yet studies rarely examine culture’s interaction with other dimensions within a unified JD-R model.

Policy support operates at the macro-level. Teachers’ perceptions of equitable policy implementation, particularly work life balance and development opportunities, strongly influence well-being (Barbieri et al., 2019).

Effective policy implementation can provide teachers with the necessary resources and support to manage their workload and enhance their professional capabilities. In China’s centralized system, national policies such as “Double Reduction” promise relief but frequently encounter local implementation gaps, creating a “resource-discounting” effect (Chen and Lin, 2024; Chen, 2024).

On the demand side, Workload is he most documented stressor. Excessive hours, administrative tasks, and examination pressure erode well-being (Burns and Machin, 2013; Wang and Zhang, 2017; Zhou and Ning, 2020). Integrative reviews of Chinese teachers confirm workload as a unique cultural demand tied to exam-oriented accountability and parental expectations (Chen et al., 2022). Management Systems, including transparent policies and fair administrative practices, can either buffer or amplify strain (Day, 2017; Schnackenberg and Tomlinson, 2016).

While prior research has examined these dimensions in isolation, their synergistic and compensatory effects remain under-specified. The present study therefore operationalizes the working environment as a six-dimensional constellation and tests their relative predictive power within a single JD-R model—an approach that addresses a clear gap in both Western and Chinese literatures.

### 2.3 Contextual mediators in Chinese education

China’s educational context introduces distinct boundary conditions. Rural–urban disparities shape TOWB. Rural teachers often report higher well-being due to tighter communities, yet face resource scarcity and limited professional development (Burns and Machin, 2013; Omojemite and Cishe, 2025; Wang et al., 2025). Seniority-based hierarchies further moderate outcomes; senior teachers enjoy greater recognition, while younger colleagues experience inequity and higher stress (Zhou and Ning, 2020).

Recent reforms, notably the “Double Reduction” policy, have intensified workload paradoxes: intended to reduce student burden, they have shifted administrative and supervisory demands onto teachers, elevating burnout risk (Yongtao, 2025; Zhao and Wu, 2025). These contextual mediators highlight why de-contextualized JD-R applications risk policy misfires.

### 2.4 Research questions and hypotheses

To address the identified gaps, this study examines three research questions:

1. What is the current level of TOWB among Chinese primary school teachers, and how does it vary demographically?
2. How do the six dimensions of working environment influence TOWB?
3. Is TOWB improved when the working environment is enhanced?

Building on the synthesized literature and the conceptual model (Figure 1), we advance the following hypotheses:

*H1a*: Professional development is positively associated with TOWB.

*H1b*: Collaborative school culture is positively associated with TOWB.

*H1c*: Policy support is positively associated with TOWB.

*H2*: Perceived workload is negatively associated with TOWB.

*H3a*: A well-functioning management system is positively associated with TOWB.

*H3b*: Favorable physical and material working conditions are positively associated with TOWB.

*H4*: An enhanced working environment leads to higher levels of TOWB compared to a less favorable working environment.

These hypotheses enable a theoretically driven, empirically integrated examination of how environmental resources counteract culturally embedded demands in Chinese primary schools.

## 3 Materials and methods

This study adopts a quantitative, theory-driven design grounded in the JD-R framework. It employs a cross-sectional national survey dataset to address three research questions and hypotheses formulated in Section 2.4. The analytical sequence proceeds in four stages: (1) descriptive and preliminary analyses to map TOWB distributions and demographic patterns; (2) bivariate correlations and multivariate ordinary least squares (OLS) regression to examine direct associations between the six-dimensional working environment and TOWB; (3) CEM and SEM to verify the robustness of the OLS estimates regarding environmental enhancements.

### 3.1 Data source and sampling

The data for this study were derived from the publicly available China Teacher Development Report (CTDR) survey, conducted by Northeast Normal University in 2020 (Li and Gai, 2022). The survey employed a stratified random sampling design to ensure a representative sample of primary school teachers across China. The 31 provinces and municipalities were first stratified by region (Eastern, Central, Western, Northeastern) and school location (urban/rural). Within each stratum, schools were randomly selected, and all primary school teachers within these schools were invited to participate electronically. This stratified approach aimed to capture the diversity of teaching contexts across different regions and urban–rural settings.

Participants provided informed electronic consent before proceeding with the survey. The survey guaranteed anonymity, collecting no personally identifiable information. Ethical approval was granted by the Academic Ethics Committee of Northeast Normal University’s Faculty of Education, ensuring adherence to ethical standards, including confidentiality and voluntary participation. From the original 33,590 questionnaires, data cleaning excluded responses with completion times under 15 min or identical answers across all items. The final analytic sample comprised 25,028 primary school teachers, representing a valid response rate of 92.67%. Detailed information on the study subjects is shown in Table 1.

**Table 1:** Respondents’ distribution.

**Category**	**Items**	**Frequency**	**Percentage(%)**
Gender	Male	5,050	20.18
	Female	19,978	79.82
Marital status	Married	19,877	79.42
	Single	5,151	20.58
Years of teaching	0 ~ 2 years	3,542	14.15
	3 ~ 5 years	2,904	11.60
	6 ~ 10 years	3,103	12.40
	11 ~ 15 years	1,902	7.60
	16 ~ 20 years	2,708	10.82
	21 ~ 25 years	4,387	17.53
	26 ~ 30 years	2,893	11.56
	31 ~ 35 years	2,318	9.26
	over 36 ~ 40 years	1,271	5.08
Professional title	ungraded	3,913	15.63
	Level III	478	1.91
	Level II	6,675	26.67
	Level I	10,338	41.31
	Senior	3,624	14.48
Urban/rural	Urban	11,414	45.60
	Rural	13,614	54.40
Regions	Eastern	2,087	8.34
	Central	4,097	16.37
	Western	9,020	36.04
	Northeastern	9,824	39.25

### 3.2 Measures

#### 3.2.1 TOWB scale validation

TOWB was measured using the validated and psychometrically robust Structure Questionnaire of Teachers’ Occupational Well-being developed by Li and Gai (2022). This scale, developed and validated specifically within the Chinese educational context, demonstrated strong construct validity through Confirmatory Factor Analysis (CFA: CFI = 0.96, TLI = 0.95, RMSEA = 0.04) and high internal consistency in its original validation study. It comprises four dimensions measured on a 5-point Likert scale (1 = Strongly Inconsistent, 5 = Strongly Consistent).

Subjective well-being was determined using nine items designed to reflect teachers’ satisfaction and sense of accomplishment with their current job and teaching profession. Examples of items included “You are satisfied with your performance in school” and “The value of your life has been well reflected in the work.” The internal consistency reliability, as measured by Cronbach’s alpha, was 0.94, indicating high reliability. Cognitive well-being was measured using 20 items aimed at capturing teachers’ competency and their sense of occupational security and attraction. Examples of items included “You are flexible to evaluate student development in a variety of ways” and “Teaching has become an enviable profession in your region.” Cronbach’s alpha was 0.89, indicating good reliability.

Physical and mental well-being was determined using nine items that assess teachers’ physical and mental health situation. Examples of items included “You often do not get efficient sleep because of your job” and “You often feel anxious and tired at work.” Cronbach’s alpha, was 0.87. Social well-being was assessed using 17 items aimed at determining teachers’ social interactions with their students, colleagues, leaders, and parents. Examples of items included “You always get help from colleagues when you encounter difficulties” and “Students enjoy being with you and often invite you to participate in their activities.” And Cronbach’s alpha was 0.94.

#### 3.2.2 Working environment instrument

The working environment scale was developed by the CTDR research team based on extensive literature review and pilot interviews to ensure contextual relevance. It consists of 27 items measuring six dimensions on a 5-point Likert scale (1 = Strongly Inconsistent, 5 = Strongly Consistent), with higher scores indicating a more favorable environment.

The six dimensions are: (1)working conditions (e.g., “Office conditions can meet your work needs”), (2)workload (e.g., “You have enough time to complete expected work”), (3)professional development (e.g., “There is frequent teaching and research activities which provide all kinds of development paths for teachers at different stages of development”), (4)school culture (e.g., “Your school managers pay attention to the culture construction of disciplinary team”), (5)management system (e.g., “There are sound school systems in your school”), and (6)policy support (e.g., “With the support of national policies, you are positive about the career development prospect”). The overall scale demonstrated good internal consistency (*α* = 0.87). While developed for this survey, its face validity and content validity were established through expert review and pilot testing. EFA conducted on the CTDR dataset supported the six-factor structure (results available upon request).

### 3.3 Analytical procedures

#### 3.3.1 Preliminary analyses (ANOVA)

To ensure that variables of interest did not vary significantly across different cohorts, we conducted an initial analysis. The results indicated significant differences among cohorts in several key demographic and professional characteristics, including regions, location, gender, marital status, professional title, years of teaching, and school type. This step was crucial to identify any potential confounding factors that might influence the subsequent analyses.

#### 3.3.2 Multivariate regression models

To answer Research Question 2 and test Hypotheses H1a–H4, we first examined the relations between working environment and TOWB using bivariate correlations. This approach allowed us to understand the direct associations between these variables without the influence of other factors. After confirming OLS assumptions, we used multivariate ordinary least squares (OLS) regression following the procedure outlined by Aiken (1991). TOWB served as the dependent variable, while the six working-environment dimensions were entered as independent variables. All continuous predictors were mean-centered prior to analysis to reduce multicollinearity and facilitate interpretation of interaction terms. Variance Inflation Factors (VIF) were below 5 and tolerance values exceeded 0.2, confirming no problematic multicollinearity. To achieve net influencing results of the working environment, we controlled for school region, location, teacher’s gender, marital status, teaching experiences, professional title, employment motivation, and family relationship.

#### 3.3.3 Coarsened exact matching protocol

To answer the Research Question 3 and provide stronger evidence regarding Hypotheses H4, we adopted a quasi-experimental design based on CEM method (Blackwell et al., 2009). CEM works by exact matching on distilled information in the covariates as chosen. Firstly, we took the overall working environment score as the treatment variable and divided the sample teachers into the treatment group (Treat = 1) with scores above the median (3.579) and the control group (Treat = 0) with scores below the median. Secondly, we controlled for a set of pretreatment variables (gender, age, years of teaching, professional title) by the CEM algorithm. Finally, we estimated the average treated effect on the treated (ATT) through multiple regression models with CEM weights in STATA.

Despite improved balance (L1 reduced to 0.038), the cross-sectional design precludes definitive causality. Therefore, results should be interpreted as robust evidence consistent with causal effects rather than definitive proof of causality. Since the cross-sectional design of the CTDR data precludes ruling out reverse causality, future longitudinal or quasi-experimental designs are needed to establish temporal precedence.

#### 3.3.4 Supplementary SEM

To provide a comprehensive test of the overall conceptual model (Figure 1) and examine potential mediation among the six working-environment dimensions, we conducted supplementary SEM analysis using STATA’s sem command. The model treated the six dimensions as observed predictors of latent TOWB (four first-order factors loading onto a second-order well-being construct). Maximum-likelihood estimation with robust standard errors was employed to handle non-normality. Model fit was evaluated using standard indices (CFI ≥ 0.95, TLI ≥ 0.95, RMSEA ≤ 0.06, SRMR ≤ 0.08).

## 4 Results

### 4.1 Descriptive and demographic patterns

The analysis indicates that TOWB remains at a moderate overall level, suggesting substantial room for improvement within the current system. Across demographic groups, statistically significant variations are observed, indicating that TOWB is not uniformly distributed but shaped by structural and individual characteristics.

Specifically, female teachers report higher TOWB (*M* = 3.721, SD = 0.004) than male teachers (*M* = 3.587, SD = 0.009) (*t* = 2.094, *p* < 0.001). Regional disparities are also evident, with teachers in the Northeastern region reporting the highest TOWB (*M* = 3.793, SD = 0.595), compared to the Western region (*M* = 3.630, SD = 0.594) [*F*(3, 25,024) = 126.86, *p* < 0.001]. In addition, TOWB increases with years of teaching experience, with teachers having over 35 years of experience reporting the highest levels (*M* = 4.010, SD = 0.568). Similarly, senior title holders exhibit higher TOWB (*M* = 4.073, SD = 0.475) than lower-ranked teachers [*F*(5, 25,022) = 118.16, *p* < 0.001]. These patterns suggest that both institutional positioning and career-stage factors systematically relate to variations in TOWB, thereby providing an important baseline for subsequent multivariate analyses addressing Research Question 1.

Rural teachers exhibited significantly higher TOWB (*M* = 3.761, SD = 0.005) than urban counterparts (*M* = 3.674, SD = 0.005; *t* = 11.668, *p* < 0.001). This finding indicates that contextual features of school environments may play a role in shaping well-being beyond individual characteristics. It further underscores the need to examine working-environment factors in an integrated framework.

### 4.2 Associations between working environment and TOWB

Bivariate correlation analysis (see Table 2) indicated strong positive correlations between the overall working environment and TOWB (*r* = 0.80, *p* < 0.001), providing initial support for the expectation that more favorable environments are linked to higher levels of well-being (Research Question 2).

**Table 2:** Means, standard deviations, and correlations between study variables (*N* = 25,028).

Variables	Means	SD	Cor
TWOB	3.72	0.59	
Subject wellbeing	3.77	0.68	
Cognitive wellbeing	3.75	0.54	
Physical and mental wellbeing	3.15	0.84	
Social wellbeing	3.82	0.66	
Working environment	3.60	0.68	0.80^∗∗∗^
Working conditions	3.69	0.95	0.60^∗∗∗^
Workload	2.04	0.90	−0.56^∗∗∗^
Professional development	3.80	0.69	0.79^∗∗∗^
School culture	4.04	0.76	0.71^∗∗∗^
Management system	3.64	0.81	0.70^∗∗∗^
Policy support	3.53	0.76	0.71^∗∗∗^

^∗∗∗^*p* < 0.001.

Among the six dimensions, professional development (*r* = 0.79), school culture (*r* = 0.71), management system (*r* = 0.70), and policy support (*r* = 0.71) are strongly positively associated with TOWB (all *p* < 0.001), whereas workload is negatively associated (*r* = −0.56, *p* < 0.001). These patterns are broadly consistent with Hypotheses H1a–H1c, suggesting that job resources are positively related to TOWB, while job demands show an inverse relationship.

Regarding organizational conditions, both management system (*r* = 0.70, *p* < 0.001) and working conditions (*r* = 0.60, *p* < 0.001) are positively correlated with TOWB. These associations provide initial support for H3a and H3b, suggesting that well-structured institutional arrangements and favorable physical environments are related to improved well-being at the bivariate level.

### 4.3 Multivariate regression outcomes

Multivariate OLS regression tested the net effects of the six working-environment dimensions on TOWB while controlling for all demographic and school-level covariates (Table 3, Model 3). The full model explained 71.6% of the variance in TOWB [Adj. *R*^2^ = 0.783, *F*(13, 25,01) = 6,957.62, *p* < 0.001]. Professional development (*β* = 0.262, *p* < 0.001), school culture (*β* = 0.07, *p* < 0.001) and policy support (*β* = 0.153, *p* < 0.001) showed significant positive effects. Then H1a, H1b and H1c were supported. While perceived workload exerted a significant negative effect (*β* = −0.009, *p* < 0.001), suggesting H2 was supported. In terms of organizational conditions, management system (*β* = 0.014, *p* < 0.001) showed positive associations, suggesting H3a was supported. While working conditions were not significant once other variables were controlled (*β* = 0.0001, *p* > 0.05). Then H3b was not supported.

**Table 3:** Results from multivariate OLS regression and CEM.

**Variables**	**Model 1**		**Model 2**		**Model 3**		**Model 4**	
	***B***	**SE**	***B***	**SE**	***B***	**SE**	***B***	**SE**
Working environment	0.697^∗∗∗^	0.003						
Working conditions			0.030^∗∗∗^	0.003	0.002	0.003	0.0001	0.003
Workload			−0.135^∗∗∗^	0.003	−0.099^∗∗∗^	0.002	−0.111^∗∗∗^	0.002
Professional development			0.362^∗∗∗^	0.007	0.262^∗∗∗^	0.006	0.264^∗∗∗^	0.006
School culture			0.143^∗∗∗^	0.005	0.070^∗∗∗^	0.005	0.054^∗∗∗^	0.004
Management system			0.016^∗∗∗^	0.005	0.014^∗∗∗^	0.004	0.012^∗∗∗^	0.004
Policy support			0.187^∗∗∗^	0.004	0.153^∗∗∗^	0.003	0.156^∗∗∗^	0.003
Regions					0.018^∗∗∗^	0.002	0.022^∗∗∗^	0.002
Urban					−0.036^∗∗∗^	0.004	−0.036^∗∗∗^	0.004
Gender					0.024^∗∗∗^	0.004	0.021^∗∗∗^	0.005
Years of teaching					0.018^∗∗∗^	0.001	0.022^∗∗∗^	0.001
Professional title					−0.019^∗∗∗^	0.002	−0.024^∗∗∗^	0.002
Class size					−0.021^∗∗∗^	0.001	−0.021^∗∗∗^	0.002
Characteristics					0.298^∗∗∗^	0.004	0.323^∗∗∗^	0.004
*R*2	0.648		0.713		0.783		0.716	
Adj. *R*2	0.648		0.713		0.783		0.715	
*F*	(1,2,502) 46,954.17		(6,2,502) 10,344.05		(13,2,501) 6,957.62		(13,2,438) 4717.45	
*N*	25,028		25,028		25,028		24,395	

Dependent variable: TOWB. TOWB, working environment and its each dimension, personal factors were centered at their means. ∗*p* < 0.05; ∗∗*p* < 0.01; ∗∗∗*p* < 0.001.

### 4.4 Robust test via CEM and SEM

To verify the robustness of the OLS estimates, we conducted both CEM and SEM. Using CEM, we balanced pretreatment covariates (gender, age, teaching years, professional title), reducing multivariate imbalance from L1 = 0.384 to 0.038 (Table 4). The ATT estimates (Table 3, Model 4) confirmed the OLS findings. Professional development, policy support, and workload remained the strongest predictors. In parallel, SEM with TOWB specified as a second-order latent construct showed excellent model fit (CFI = 0.96, TLI = 0.95, RMSEA = 0.045). As reported in Table 5, the direct effects of all six dimensions were nearly identical to the OLS coefficients. These convergent results from two distinct methods confirm that the multivariate findings are robust and not artefacts of measurement error or covariate imbalance.

**Table 4:** Imbalance measured results of CEM.

Matching Status	Variable	L1	Mean	Min	25%	50%	75%	Max
Before matching	Gender	0.008	0.008	0	0	0	0	0
	Years of teaching	0.073	−0.012	0	−1	0	0	0
	Professional title	0.090	−0.057	0	0	0	1	0
	Characteristics	0.347	0.515	0.5	0.5	0.75	0	0
After matching	Gender	2.6E-15	8.0E-15	0	0	0	0	0
	Years of teaching	5.9E-15	−3.8E-14	0	0	0	0	0
	Professional title	6.4E-15	−3.0E-14	0	0	0	0	0
	Characteristics	0.025	0.006	0	0	0	0	0

**Table 5:** Direct effects of SEM (*N* = 25,028).

**Path**	**B**	**SE**	**95% CI**
Working conditions	0.001	0.003	[−0.005, 0.007]
Workload	−0.098^∗∗∗^	0.002	[−0.102, −0.094]
Professional development	0.260^∗∗∗^	0.006	[0.248, 0.272]
School culture	0.069^∗∗∗^	0.005	[0.059, 0.079]
Management system	0.015^∗∗∗^	0.004	[0.007, 0.023]
Policy support	0.155^∗∗∗^	0.004	[0.147, 0.163]
CFI = 0.96; TLI = 0.95; RMSEA = 0.045 (90% CI [0.042, 0.048]); SRMR = 0.038			

All continuous variables were mean-centered prior to analysis. Maximum-likelihood estimation with robust standard errors was used. TOWB was modelled as a second-order latent construct with four first-order dimensions (subjective, cognitive, physical/mental, and social well-being).

## 5 Discussion

### 5.1 Key findings revisited

The present study addressed three research questions derived from the JD-R framework (Figure 1). First, Chinese primary school teachers reported a moderate overall level of TOWB, with notable demographic variations. Female, rural, more experienced, and senior-title teachers exhibited higher scores. Second, the six-dimensional working environment emerged as a powerful predictor, explaining 71.6% of TOWB variance in the fully controlled OLS model and showing excellent fit in SEM analysis. Professional development and policy support exerted the strongest positive effects, workload the strongest negative effect, while school culture and management system provided smaller but significant positive contributions. Third, CEM and SEM provided convergent evidence consistent with a positive effect of an enhanced working environment on TOWB. These results provide coherent empirical support for the hypothesized model and suggest that TOWB is shaped by a structured configuration of environmental conditions rather than isolated factors.

### 5.2 Theoretical integration

The findings provide a refined elaboration of the JD-R model in a non-Western, high-stakes primary education context. Professional development emerged as the dominant job resource, consistent with the autonomy-fulfilment mechanism. When teachers gain access to continuous, stage-appropriate learning opportunities, they experience heightened professional agency and efficacy, which in turn buffer emotional exhaustion and foster engagement (Chen et al., 2022; Hollar et al., 2022).

Policy support functioned as a macro-level resource whose effect was partly transmitted via management systems (Bakker and Demerouti, 2017), aligning with the resource-discounting hypotheses. In China’s centralized system, national policies promise relief but lose potency when implementation is inconsistent; the present data thus highlight a boundary condition of JD-R theory—formal policy resources require local fidelity to realize their buffering potential.

Conversely, workload operated as a classic job demand, exerting a robust negative direct effect that was not fully offset by resources. This pattern supports the demand-threat mechanism: excessive administrative and examination-related tasks deplete cognitive and emotional reserves, leading to lower subjective, physical/mental, and social well-being. The magnitude of standardized coefficients for the three resource dimensions (ranging from *β* = 0.054 to 0.264) collectively outweighs the single demand dimension (*β* = −0.111), consistent with the JD-R model’s prediction that resources buffer the negative impact of demands. Future research should include multiple demand dimensions to enable a more balanced test.

### 5.3 Chinese contextualization

Several findings are deeply rooted in China’s educational realities. Rural teachers’ higher TOWB, despite resource constraints, reflects the protective effect of smaller, more cohesive school communities, while urban teachers appear more vulnerable to performative pressures. Seniority-based hierarchies similarly amplify disparities: senior teachers benefit from recognition and reduced teaching loads, whereas early-career colleagues face inequity. The “Double Reduction” policy paradox is particularly instructive—intended to alleviate student burden, it inadvertently shifted administrative and supervisory demands onto teachers (Chen, 2024; Zhao and Wu, 2025), illustrating how well-intentioned macro reforms can intensify workload without corresponding resource enhancements. These contextual mediators underscore why de-contextualized JD-R applications risk policy misfires and reinforce the necessity of culturally attuned operationalizations. Alternatively, it is possible that rural teachers have lower expectations or different reference points for well-being, which may suppress the negative effects of material scarcity. This alternative hypotheses warrants future mixed-methods investigation.

### 5.4 Practical implications

The findings generate several actionable implications for both school-level leadership and policy design.

At the school level, enhancing TOWB requires systematic investment in professional development as a core organizational function, rather than treating it as a peripheral activity. Schools should also foster collaborative and supportive cultures that enable teachers to share practices and reduce professional isolation.

At the policy level, reducing workload should be prioritized through institutional mechanisms such as workload auditing and administrative streamlining. In addition, improving the consistency and transparency of policy implementation is essential to ensure that formal support translates into perceived benefits for teachers.

Importantly, these interventions should be designed as integrated strategies, as the results indicate that isolated improvements are less effective than coordinated changes across multiple dimensions of the working environment.

### 5.5 International relevance

Although grounded in the Chinese context, the study’s core findings resonate with global research on TOWB. Workload remains a universal threat across diverse systems (Akyurek and Can, 2022; OFSTED, 2019), while professional development and supportive school cultures consistently emerge as protective resources (Thien and Lee, 2023). The resource-discounting effects echo recent international calls for integrated, multi-level interventions rather than single-factor fixes (Li et al., 2025). At the same time, China’s experience offers distinctive lessons for other high-accountability, centralized systems: the implementation gap between national policy rhetoric and local practice is not unique, yet the scale of the Chinese dataset and the explicit testing of six inter-related dimensions provide a replicable template for cross-national comparative work. Future international studies could usefully examine whether similar synergistic patterns hold in decentralized or low-stakes contexts, thereby refining the boundary conditions of the JD-R model.

## 6 Conclusion

### 6.1 Theoretical contributions

This study makes three substantive advances to the literature on teacher occupational well-being. First, it provides a contextual extension of the JD-R model by operationalizing the working environment as a culturally attuned, six-dimensional constellation and testing its predictive power in a large, nationally representative sample of Chinese primary school teachers operating under examination-oriented pressures. Second, it offers a methodological contribution by integrating multivariate OLS regression, CEM, and supplementary SEM on the same dataset. This multi-method approach not only establishes robust associations but also generates evidence consistent with causal improvement when the working environment is enhanced while accounting for measurement error and latent structure in the SEM component. Third, it delivers a conceptual refinement by demonstrating the synergistic buffering role of job resources (particularly professional development and policy support), the demand-threat mechanism of workload, and the partial mediation effects. These findings clarify boundary conditions of the JD-R framework in non-Western, high-accountability systems and move the field beyond single-factor studies toward a more integrative, multi-level understanding of person–environment dynamics.

### 6.2 Practical innovations

The results translate directly into actionable levers for both school leaders and national policymakers. School principals should prioritize sustained, teacher-agency-oriented professional development programs and deliberate culture-building initiatives that capitalize on the strongest positive predictors identified. Concurrently, workload audits and streamlined administrative protocols are essential to address the most potent negative driver. At the policy level, the Chinese government and local education bureaus should focus on closing implementation gaps. Targeted support packages for urban and early-career teachers could further reduce well-being disparities. These innovations, grounded in empirical evidence from 25,028 teachers, offer a replicable blueprint that can be adapted by education systems worldwide seeking to enhance teacher retention and instructional quality.

### 6.3 Limitations and future research

Several limitations should be acknowledged. First, although the CTDR dataset is large and nationally representative, the cross-sectional design precludes definitive causal inference; CEM and SEM strengthen the evidence but cannot fully substitute for longitudinal or experimental data. Second, all measures rely on self-reports, raising the possibility of common-method bias and social-desirability effects. Third, the findings are embedded in the specific cultural and structural features of China’s examination-oriented primary education system; generalizability to other national contexts, school levels, or decentralized systems requires caution. Fourth, the absence of objective indicators limits triangulation of subjective perceptions.

Future research should address these gaps through longitudinal designs that track changes in TOWB over time and evaluate the sustainability of environmental interventions. Mixed-methods studies combining quantitative scales with in-depth interviews and case studies would illuminate the lived mechanisms underlying the identified pathways. Comparative cross-national investigations could test whether the six-dimensional model and mediation effects replicate in low-stakes or decentralized systems. Finally, intervention studies that experimentally manipulate professional development, workload reduction, or policy implementation fidelity would provide the strongest test of the practical innovations proposed here. By pursuing these directions, scholars and policymakers can continue refining strategies to support teacher well-being and, ultimately, improve educational outcomes for students worldwide.

## Data Availability

The dataset is publicly available as part of the China Teacher Development Report (CTDR) survey. The data were accessed and analyzed in accordance with the terms of use provided by Northeast Normal University. Detailed information regarding the survey methodology and access procedures is described in the following publication: Li and Gai (2022). Inquiries about data access can be directed to the corresponding author.
